# Selection of viral variants during persistent infection of insectivorous bat cells with Middle East respiratory syndrome coronavirus

**DOI:** 10.1038/s41598-020-64264-1

**Published:** 2020-04-29

**Authors:** Arinjay Banerjee, Sonu Subudhi, Noreen Rapin, Jocelyne Lew, Richa Jain, Darryl Falzarano, Vikram Misra

**Affiliations:** 1 Department of Veterinary Microbiology, Western College of Veterinary Medicine, University of Saskatchewan, Saskatoon, SK Canada; 2 Vaccine and Infectious Disease Organization-International Vaccine Centre (VIDO-InterVac), Saskatoon, SK Canada; 3 Present Address: Department of Pathology and Molecular Medicine, Michael DeGroote Institute for Infectious Disease Research, McMaster Immunology Research Centre, McMaster University, Hamilton, ON Canada; 4 Present Address: Gastrointestinal Unit and Liver Center, Massachusetts General Hospital, Harvard Medical School, Boston, MA USA

**Keywords:** Biological techniques, Cell biology, Evolution, Microbiology, Molecular biology

## Abstract

Coronaviruses that cause severe acute respiratory syndrome (SARS) and Middle East respiratory syndrome (MERS) are speculated to have originated in bats. The mechanisms by which these viruses are maintained in individuals or populations of reservoir bats remain an enigma. Mathematical models have predicted long-term persistent infection with low levels of periodic shedding as a likely route for virus maintenance and spillover from bats. In this study, we tested the hypothesis that bat cells and MERS coronavirus (CoV) can co-exist *in vitro*. To test our hypothesis, we established a long-term coronavirus infection model of bat cells that are persistently infected with MERS-CoV. We infected cells from *Eptesicus fuscus* with MERS-CoV and maintained them in culture for at least 126 days. We characterized the persistently infected cells by detecting virus particles, protein and transcripts. Basal levels of type I interferon in the long-term infected bat cells were higher, relative to uninfected cells, and disrupting the interferon response in persistently infected bat cells increased virus replication. By sequencing the whole genome of MERS-CoV from persistently infected bat cells, we identified that bat cells repeatedly selected for viral variants that contained mutations in the viral open reading frame 5 (ORF5) protein. Furthermore, bat cells that were persistently infected with ΔORF5 MERS-CoV were resistant to superinfection by wildtype virus, likely due to reduced levels of the virus receptor, dipeptidyl peptidase 4 (DPP4) and higher basal levels of interferon in these cells. In summary, our study provides evidence for a model of coronavirus persistence in bats, along with the establishment of a unique persistently infected cell culture model to study MERS-CoV-bat interactions.

## Introduction

On rare occasions, viruses spill over from reservoir species to other animals, including humans^1^. Establishment of infection in the new host requires viruses to adapt to the efficient use of entry receptors and circumvent innate antiviral defense mechanisms that are unique to each host species^2^. The elaborate mechanisms underlying such changes that govern new virus-host dynamics are not well known. Bats are speculated to be reservoirs of several emerging viruses, including coronaviruses (CoVs) that cause severe acute respiratory syndrome (SARS) and Middle East respiratory syndrome (MERS) in humans, and porcine epidemic diarrhea (PED) and swine acute diarrhoea syndrome (SADS) in pigs^3–6^. Although bats harbor SARS- and MERS-like coronaviruses, overt signs of disease in bats that are naturally or experimentally infected are often undetectable^7^. In contrast, infections in spillover species, such as humans and pigs lead to diseases with high morbidity and mortality^7–13^.

MERS-CoV is an on-going concern as it causes periodic outbreaks in the Middle East with a mortality rate of about thirty-five percent^14,15^. Human-to-human transmission of the virus occurs through aerosol or close contact. Camels are the known reservoirs of MERS-CoV^16,17^ and bats are suspected to be the ancestral host^18^. MERS-CoV belongs to the betacoronavirus lineage 2c. Multiple 2c coronaviruses, such as HKU4, HKU5 and NeoCoV have been detected in bats^19,20^. Coronaviruses, such as MERS-CoV rapidly adapt to the species they infect. These adaptations usually occur in the spike protein as its interaction with the host receptor is necessary for infection^2,21^. Although bats are speculated as the ancestral host of MERS-CoV, host-specific adaptations across the MERS-CoV genome after propagation in bats or bat cells have not been extensively investigated.

Much of the information about MERS-CoV are derived from observations in human or rodent cells and rodent or camelid animal models^22–24^. Little is known about long-term molecular processes that govern the relationship of the virus with its ancestral bat host. A recent study showed that bat CoVs can persist in their natural bat host for at least four months of hibernation^25^. The authors observed that persistent infection is disrupted by immunologically stressful events, such as a secondary fungal infection with the white-nose syndrome causing fungus, *Pseudogymnoascus destructans* leading to an increase in virus replication^26^. Persistence of viruses in bats and increase in virus shedding during ‘stressful’ events has also been observed with Henipavirus infections^27,28^.

Members of the coronavirus family possess high levels of genetic variability, especially in coding sequences for their accessory proteins, but not in the conserved viral polymerase^18^. Accessory proteins are dispensable for virus replication but provide vital functions within the context of infection in a particular host. Primarily, these proteins modulate host antiviral responses against invading coronaviruses^24^. MERS-CoV accessory proteins, ORFs 4a and 4b can inhibit type I interferon (IFN) response^24^ and ORF5 has been shown to modulate the NF-κB pathway^29^. Despite these studies in cultured human cell lines, cellular interactions of MERS-CoV accessory proteins in bat cells are unknown.

As insectivorous bats are speculated as the ancestral hosts for coronaviruses^18^, studying the mechanisms that lead to persistent infections in bats will provide some clues about how bats can harbor different coronaviruses. In this study, we have identified coronavirus and host factors that enable a long-term persistent infection in cultured bat cells. We show for the first time that cells from an insectivorous bat, *Eptesicus fuscus*, can be persistently infected with MERS-CoV over a period of several months. Disrupting interferon regulatory factor 3 (IRF3)-mediated antiviral signaling pathways and inhibiting MAP kinase pathways in these cells lead to an increase in virus replication. In addition, the establishment of persistent infection in bat cells in two separate experiments consistently identified inactivating mutations in the viral *ORF5* gene. Data from our study provide a holistic view of virus-host interactions in a unique, long-term MERS-CoV persistent infection model of bat cells.

## Results

### MERS-CoV persistently infects insectivorous bat cells

**Figure 1:**
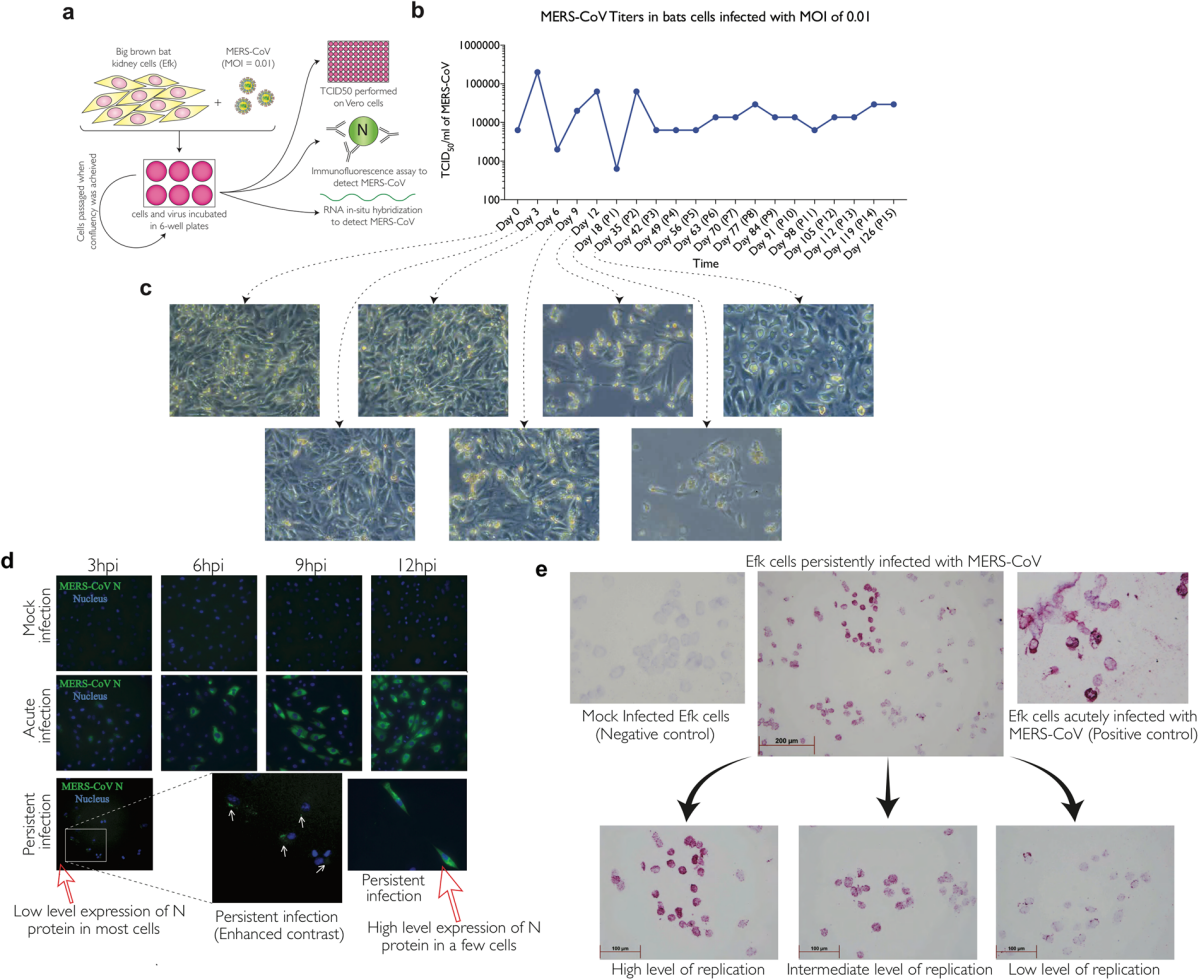
Bat cells can be persistently infected with MERS-CoV. (**a**) Big brown bat kidney cells (Efk) were infected with MERS-CoV (MOI = 0.01 TCID_50_/cell) for 12 days and then passaged weekly. Supernatant was collected during each passage to determine the presence of virus by titration on Vero cells, along with immunofluorescent and electron microscopic studies of infected cells. (**b**) Levels of MERS-CoV at different times following initial infection. (**c**) Phase contrast micrographs showing cytopathic effects on MERS-CoV infection and subsequent recovery of Efk cells at various time points. (**d**) Immunofluorescent images showing MERS-CoV nucleocapsid (N) protein in persistently infected Efk cells (bottom row; red arrows). The contrast for persistently infected Efk cells (inset) was adjusted to visualize low levels of protein. High MOI acute infection (middle row) and mock infection of Efk cells (top row) were used as positive and negative controls, respectively. Images were processed using ImageJ. (**e**) *In-situ* hybridization to detect the presence of MERS-CoV nucleoprotein RNA in persistently infected Efk cells. High, intermediate and low levels of MERS-CoV nucleoprotein RNA have been shown in the insets. Acutely infected (right) and mock infected (left) cells were used as positive and negative controls, respectively.

To investigate if MERS-CoV can persistently infect cells from an insectivorous bat, we infected big brown bat (*Eptesicus fuscus*) kidney cells (Efk)^30^ at a low multiplicity of infection (MOI; 0.01 TCID_50_/cell) (Fig. 1a). Twelve days after infection, most cells were dead (Fig. 1c). At 35 days post infection, surviving cells that were passaged formed a monolayer of continuously growing cells. We passaged the surviving cells and quantified the amount of virus in the supernatant by titration every week for 126 days (Fig. 1b). During the first 35 days of infection, the amount of virus in the supernatant of persistently infected bat cells was variable (Mean = 2.98 × 10^4^; coefficient of variation = 105.4%) but after the 42^nd^ day, we detected relatively low, but stable amounts of infectious virus in the cell culture supernatant (Mean = 1.5 × 10^4^; coefficient of variation = 58.8%) (Fig. 1b). Virus-like electron dense particles were also detected in these cells by electron microscopy (see Supplementary Fig. S1). In comparison, human lung cells (MRC5) that have been shown to support MERS-CoV infection^31^ could not be persistently infected with MERS-CoV. All cultured cells were dead several days after infection (data not shown).

### MERS-CoV N protein and RNA are detectable in persistently infected bat cells

To determine if all bat cells in culture were persistently infected, or whether persistence was maintained by small numbers of lytically-infected cells producing virus to subsequently infect susceptible cells, we analyzed these cells for the expression of MERS-CoV nucleo- (N) protein and transcripts. All persistently-infected bat cells expressed varying levels of N protein (Fig. 1d). Consistent with our observations from immunofluorescent microscopy, we detected varying levels of transcripts for MERS-CoV N in all cells in the persistently infected culture by *in-situ* hybridization (Fig. 1e).

### MERS-CoV ORF5 gene segments are undetectable in persistently infected bat cells

**Figure 2:**
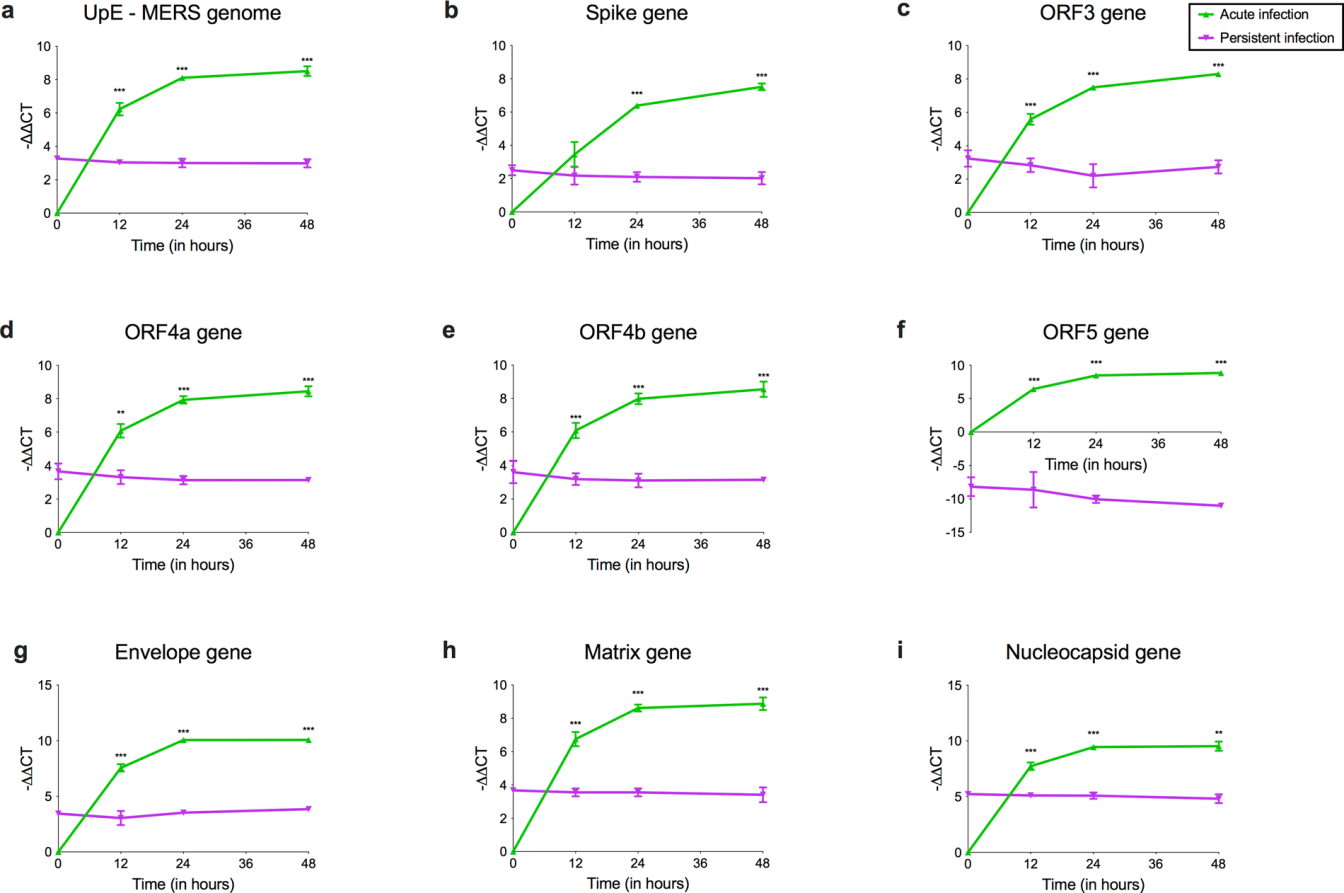
MERS-CoV gene expression varies between acute and persistently infected bat cells. RNA from persistently infected Efk cells and acutely infected Efk cells were harvested at several time points and MERS-CoV genome quantities (upE levels) and gene expression levels were analyzed by real time quantitative PCR. (**a–i)** Genome and gene expression (−(ΔCT_gene_ − ΔCT_GAPDH_)) levels for MERS-CoV upE, S, ORF3, ORF4a, ORF4b, ORF5, E, M and N genes in acute (green) vs. persistent (purple) infections at 0, 12, 24 and 48 hours post-infection or seeding, respectively (n = 4; Mean ± SD). ***P < 0.0001 and **P < 0.001 (Holm-Sidak t test with α=0.05). n = number of biological replicates.

Persistent infection is often accompanied by altered levels of virus and host gene expression, where the virus and host establish a delicate balance between cytolytic viral factors and host defensive responses^32^. To determine if there were differences in MERS-CoV replication and gene expression between acute and persistently infected bat cells, we infected bat cells with a high MOI of MERS-CoV (acute infection) and compared virus replication and gene expression in acute and persistently infected bat cells at 0, 12, 24 and 48 hours post infection (hpi) (Fig. 2a–i). For persistently infected bat cells, the time stamps in the figure indicate when RNA from these cells were harvested after seeding them in six-well plates. Expression of MERS-CoV gene segments S, E, M, N, ORFs 3, 4a, 4b and 5 increased over time in acutely infected cells (Fig. 2b–i). This was concomitant with an increase in MERS-CoV genome quantities (Fig. 2a) in acutely infected cells, as represented by upE levels^33^. In contrast, persistently infected bat cells maintained steady levels of genome (upE) quantities and with the exception of ORF5, other viral genes maintained detectable but stable levels of expression (Fig. 2a–i). We were unable to detect transcripts for ORF5 in persistently infected bat cells (Fig. 2f).

### MERS-CoV ΔORF5 mutants persistently infect bat cells

**Figure 3:**
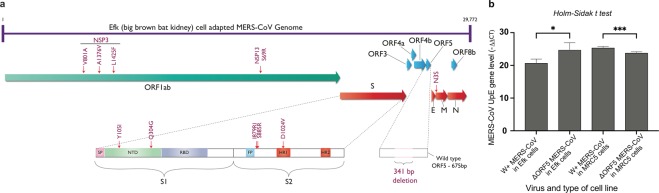
MERS-CoV ΔORF5 mutant persistently infects bat (Efk) cells. (**a**) Schematic highlighting mutations (red arrows) in the MERS-CoV genome that were identified by sequencing the dominant virus strain in persistently infected bat (Efk) cells (passage 15) are shown. (**b**) Levels of W+ and ΔORF5 MERS-CoV replication in bat (Efk) and human (MRC5) cells. Expression levels of MERS-CoV upE gene (−(ΔCT_gene_ − ΔCT_GAPDH_)), normalized to mock infected cells are shown (n = 4; Mean ± SD). *P = 0.015 and ***P = 0.0003 (Holm-Sidak t test with α = 0.05).

The MERS-CoV isolate used in our experiments (EMC/2012) was derived from an infected human and was subsequently propagated in primate cells (see materials and methods). To determine if persistently infected bat cells had selected for mutant variants of MERS-CoV, we sequenced the genome of the virus from persistently infected bat cells that had been passaged 15 times, over 4 months. We detected point mutations in the virus polymerase, spike and matrix genes (Fig. 3a) but none of these mutations disrupted the coding sequences for these proteins (Table 1). However, we observed a 341 base pair deletion in ORF5. In addition, there was a frameshift mutation at the N-terminal end of the protein that introduced a stop codon at the 20^th^ amino acid position (Fig. 3a and Table 1). In theory, the intact ORF5 coding sequence in the bat-adapted MERS-CoV strain would encode a putative 19 amino acid long protein. To identify when the ORF5 deletion mutant (ΔORF5 MERS-CoV) was selected for during the establishment of persistently infected bat cells, we sequenced ORF5 from early and late passage cells. As early as passage two, the detectable virus population in culture comprised of the ORF5 mutant. In the following portion of this article, we refer to the original human-derived virus as the MERS-CoV wildtype virus (W+) and the ORF5 deleted variant as ORF5 mutant (ΔORF5) MERS-CoV.

**Table 1:** Mutations in MERS-CoV genome rescued from persistently infected Efk cells and their possible effects on protein function.

MERS-CoV ORF	Mutations in MERS-CoV from cell line - 1	Mutations in MERS-CoV from cell line - 2	Possible effect on MERS-CoV replication and/or antiviral responses	Reference
ORFIab			1. Increased replication fidelity^67^	Graepel *et al*., 2017
NSP3	V801A	—	2. Adaptive mutations in Nsp3 to modulate bat antiviral responses	
NSP3	A1376V	—		
NSP3	L1425F	—		
NSP13	S69R	—		
NSP14	—	N373G		
Spike	Y105I	G94R	1. Increased specificity for bat Dipeptidyl peptidase 4 (DPP4)^2^	Letko *et al*., 2018; Kim *et al*., 2019; Kleine-Weber *et al*., 2018
	Q304G	V1026A	2. Increased potential to spread^68^	
	I879R	S1251F	3. Resistance to antibody-mediated neutralization^69^	
	S885R			
	D1024V			
ORF5	F20*STOP*	W108*STOP*	1. Stop codon will cause early termination of translation.	Fung and Liu, 2014; Menachery *et al*., 2017
			2. Reduced apoptosis in infected cells^70^.	
			3. Reduced ability to counteract cellular antiviral responses^29^.	
Matrix	N3S	T5A	1. Reduced ability to counteract cellular antiviral responses^24^	Yang *et al*., 2013
		D73A		

Footnote: Possible phenotypic outcomes of the mutations in ΔORF5 MERS-CoV is represented in reference to published articles that have characterized these proteins in coronaviruses. NSP, non-structural protein.

To determine if the establishment of a persistent MERS-CoV infection and the subsequent selection of viral variants was reproducible, we repeated the infection of naïve Efk (bat) cells with W + MERS-CoV at a low MOI. We recovered and sequenced ORF5 coding sequences from these cells at passages 2, 3 and 4. We detected a point mutation in ORF5 as early as passage 2, which resulted in a stop codon in the coding sequence for ORF5. In theory, the stop codon would terminate the protein at the 107^th^ amino acid. We confirmed this point mutation in ORF5 by sequencing the whole genome of MERS-CoV from persistently infected bat cells at passage 15 (Table 1). As with the previous attempt to persistently infect Efk cells with MERS-CoV, we observed point mutations in genes other than ORF5. However, unlike in ORF5, the other point mutations did not disrupt the coding sequences for other proteins (Table 1). The additional point mutations were at different sites compared to changes that were observed in the first attempt (Fig. 3a and Table 1). The possible effects of these point mutations have been summarized in Table 1. To determine if the replication kinetics of ΔORF5 were different from W + MERS-CoV, we infected bat and human cells with a high MOI of W+ and ΔORF5 MERS-CoV. ΔORF5 MERS-CoV replicated better in bat cells, whereas W + MERS-CoV replicated better in human cells (Fig. 3b).

### Bat cells infected with ΔORF5 MERS-CoV express higher levels of interferon stimulated gene transcripts

**Figure 4:**
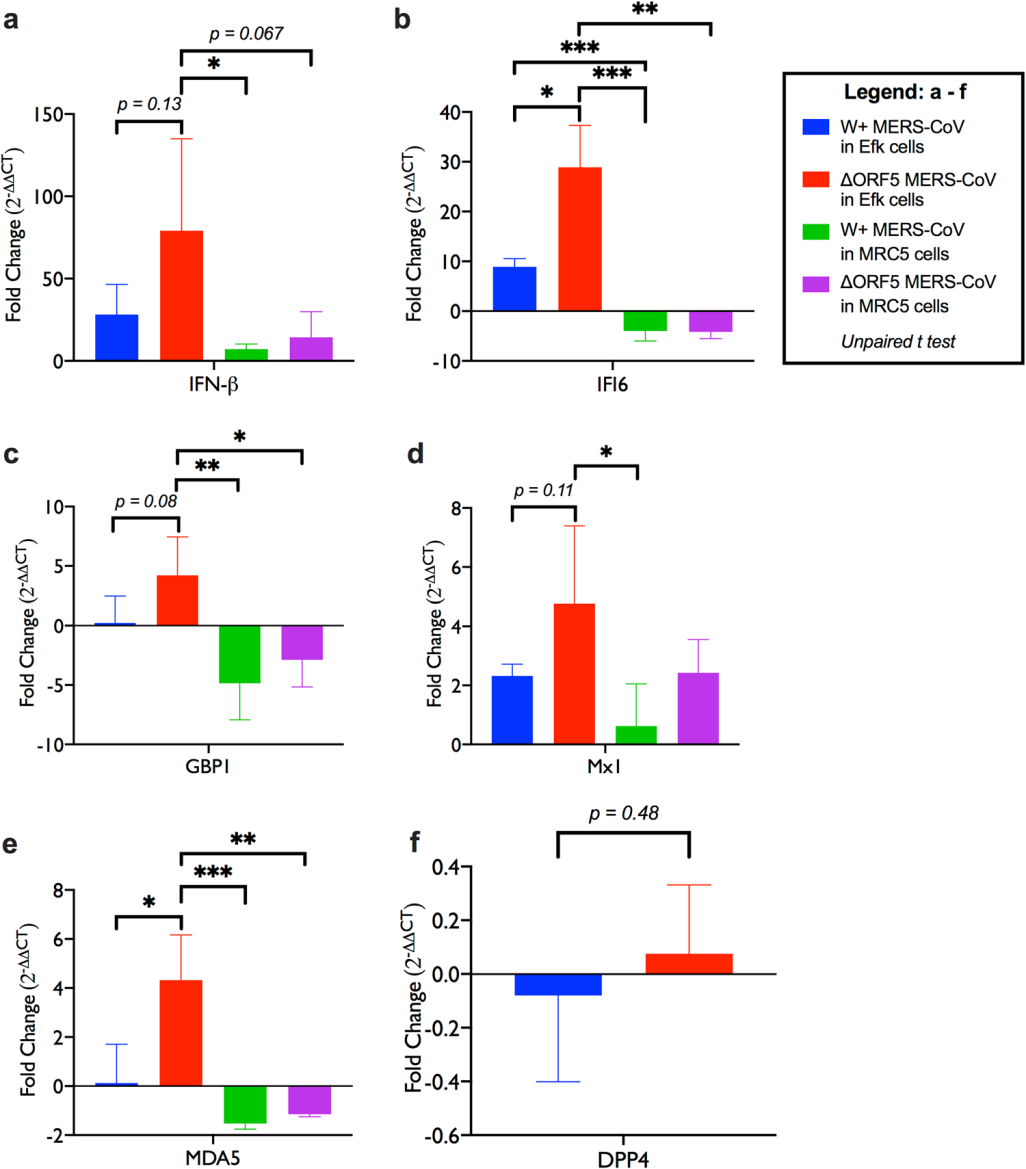
ΔORF5 MERS-CoV infection induces higher levels of IFNβ and ISGs in bat cells. (**a–e**) Transcript levels of IFNβ and interferon stimulated genes (ISGs), IFI6, GBP1, Mx1 and MDA5 in Efk and MRC5 cells infected with W+ or ΔORF5 MERS-CoV. **(f)** DPP4 transcript levels in W+ or ΔORF5 MERS-CoV infected Efk cells (n = 4; Mean ± SD). Bars represent average fold changes (2^−ΔΔCT^) in transcript levels compared to mock infected cells and normalized to GAPDH levels in each sample (n = 4; Mean ± SD). *P < 0.05; **P < 0.005; ***P < 0.0005. P values > 0.05 are mentioned in the plots (Unpaired t test with α = 0.05**)**.

A recent study demonstrated that in contrast to its ability to suppress innate antiviral responses in human cells, MERS-CoV induces a robust type I interferon response in bat cells^31^. Since ΔORF5 MERS-CoV replicated better in bat cells than W + MERS-CoV, we examined the differences in their ability to induce innate antiviral responses. We compared the levels of transcripts for interferon beta (IFNβ) and selected downstream IFN stimulated genes (ISGs), such as guanylate binding protein 1 (GBP1), myxovirus resistance 1 (Mx1), interferon regulatory factor 7 (IRF7), melanoma differentiation-associated protein 5 (MDA5) and interferon inducible protein 6 (IFI6) in human and bat cells infected with a high MOI of W+ or ΔORF5 MERS-CoV for 24 hours. IFNβ transcript levels in ΔORF5 infected bat cells were higher than W + MERS-CoV infected human cells at 24 hpi (Fig. 4a). Bat cells infected with ΔORF5 MERS-CoV contained significantly (p ≤ 0.05) higher levels of transcripts for IFI6 and MDA5 (Fig. 4b,e) than cells infected with W + virus. IFI6 transcript levels in bat cells infected with W + MERS-CoV were also significantly higher than W + MERS-CoV infected human cells (Fig. 4b). For other genes that were examined, there was an increasing trend for gene expression in bat cells infected with ΔORF5, but the differences were not statistically significant during the duration of this experiment (p = 0.08–0.11; Fig. 4c,d). There were no significant differences between human cells infected with either ΔORF5 or W + MERS-CoV.

Innate antiviral responses are induced after successful infection and detection of viral infection by cellular pattern recognition receptors (PRRs). The amount of pathogen associated molecular patterns (PAMPs), such as viral genome and transcripts determine the extent of stimulation of cellular PRRs and subsequent downstream antiviral gene expression. To determine if variation in MERS-CoV receptor levels in W+ and ΔORF5 MERS-CoV infected Efk cells could influence virus entry and subsequently, the amount of stimulatory viral genomic RNA and transcripts, we quantified the levels of MERS-CoV receptor, dipeptidyl peptidase 4 (DPP4) transcripts in bat cells infected with W+ or ΔORF5 MERS-CoV 48 hours post infection. There was no significant difference in DPP4 expression in these cells (Fig. 4f, p = 0.48).

### Persistently infected bat cells are resistant to superinfection with W + MERS-CoV

**Figure 5:**
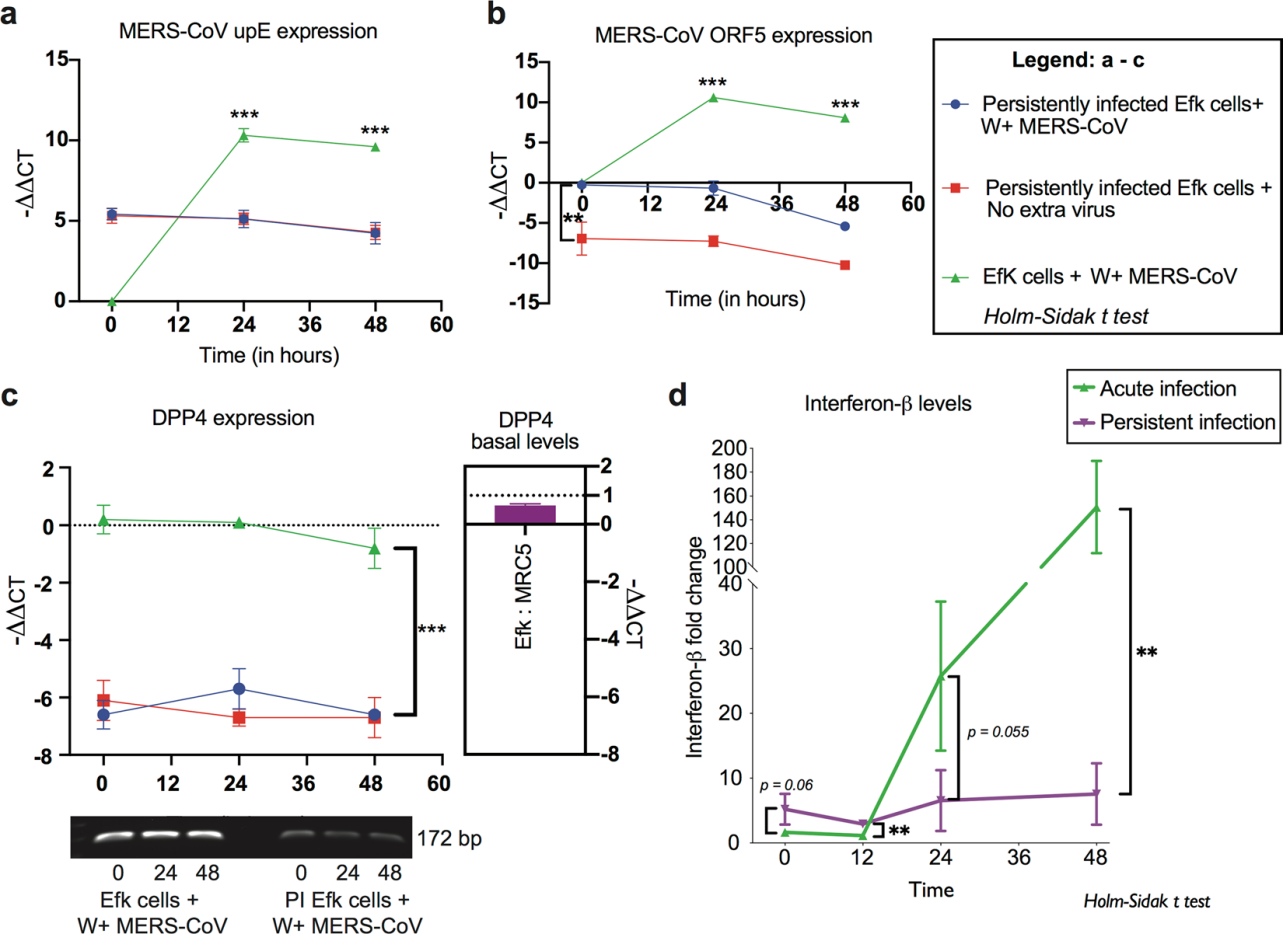
Persistently infected bat cells are resistant to super-infection with wildtype or ΔORF5 MERS-CoV. (**a–c**) Efk cells persistently infected with MERS-CoV were superinfected with W + MERS-CoV (blue) and transcript levels for (**a**) upE, (**b**) ORF5 or (**c**) *E. fuscus* dipeptidyl peptidase 4 (DPP4) were measured. The expression levels of upE, ORF5 and DPP4 transcripts (−(ΔCT_gene_ − ΔCT_GAPDH_)) were also measured in Efk cells that were persistently infected with MERS-CoV in the absence of additional virus (red) and naïve Efk cells infected with W + MERS-CoV (green), with respect to time 0 for input W + virus (n = 3; Mean ± SD). DPP4 qRT-PCR amplicons were analyzed on an agarose gel (gel inset) and a ratio of basal DPP4 transcript levels (−(ΔCT_DPP4_ − ΔCT_GAPDH_)) in naïve, uninfected Efk and MRC5 cells is shown (right panel). ***P < 0.0001 (Holm-Sidak t test with α=0.05). **(d)** IFN-β levels in persistently infected or naïve Efk cells infected with W + MERS-CoV. IFNβ fold-changes (normalized to GAPDH) are shown relative to expression levels in naïve uninfected Efk cells (n = 3; Mean ± SD). P = 0.06, 0.002, 0.055 and 0.003 at 0, 12, 24 and 48 hours post-infection/post-seeding (Holm-Sidak t test with α = 0. 05). For full size gel image in **(c)**, see supplementary Fig. S2.

Viruses often exist as quasispecies, including virus stocks that are prepared in laboratories^34^. Coronaviruses have been detected in multiple bat species globally^35^ and wild-caught bats can be persistently infected with coronaviruses^18,25^. The co-evolution of CoVs and bats, and the advantage of long-term CoV infection, if any, have not been explored in bats. In this study, we observed ΔORF5 MERS-CoV as the dominant strain in persistently infected bat cells in two independent experiments. To identify if infection with ΔORF5 MERS-CoV confers a survival advantage upon bat cells, we determined if persistently infected bat cells were resistant to superinfection with W + virus. To differentiate between W+ and ΔORF5 MERS-CoV replication, we examined the levels of genomic (upE) RNA (present in both strains), and ORF5 transcripts, (detectable in W + MERS-CoV only). On re-infecting persistently infected bat cells with W + virus (MOI = 1 TCID_50_/cell), we did not observe an increase in upE or ORF5 RNA levels in the superinfected cells, suggesting a lack of replication of W+ and ΔORF5 virus at 24 and 48 hpi (Fig. 5a,b). In contrast, we observed a significant increase in upE and ORF5 transcript levels at 24- and 48-hours post infection of naïve bat cells that were infected with an equal amount of W + MERS-CoV (Fig. 5a,b). Immediately following infection, ORF5 transcripts in superinfected bat cells (ΔORF5 + W + MERS-CoV) were significantly higher than in persistently infected bat cells (Fig. 5b), but the expression levels did not increase over time, suggesting that W + virus did not replicate and that the initial difference was due to input virus. MERS-CoV uses DPP4 as a receptor for cellular entry and both MRC5 and Efk cells contained comparable amounts of DPP4 transcripts (Fig. 5c – right panel). To determine if the persistently-infected cells were resistant to infection by W + MERS-CoV because of reduced DPP4 expression, we measured relative amounts of DPP4 transcripts in the samples described in Fig. 5a,b. Efk cells infected with W + MERS-CoV contained high levels of DPP4 transcripts, which did not decrease significantly in the 48 hrs following infection (Fig. 5c). While we could amplify DPP4 transcripts in all cells (see gel insert in Fig. 5c), in contrast to naïve Efk cells infected with W + MERS-CoV, the persistently infected cells contained almost 100 fold (average − ΔΔCT values of 6.1) less DPP4 transcripts. These levels did not change significantly in the 48 hrs following infection with W + MERS-CoV.

To determine the protective role of type I interferon in bat cells that were persistently infected with MERS-CoV, we compared the amount of IFNβ transcripts in naïve bat cells infected with W + MERS-CoV and bat cells persistently infected with ΔORF5 MERS-CoV. We observed increased levels of IFNβ transcripts in persistently infected bat cells, relative to naïve cells infected with W + virus (at 0- and 12-hours post-infection of naïve cells and post-seeding of persistently infected cells; p = 0.06 and 0.002; Fig. 5d). In addition, persistently-infected Efk cells consistently displayed higher levels of IFNβ transcripts, relative to levels in naïve, uninfected Efk cells that were used to normalize the data. As shown previously^31^, naïve Efk cells infected with W + MERS-CoV displayed a robust induction of IFNβ transcripts at 48 hpi (p = 0.003; Fig. 5d).

### Interferon regulatory factor 3 (IRF3) and mitogen-associated protein kinase (MAPK) signaling regulates persistent infection in bat cells

**Figure 6:**
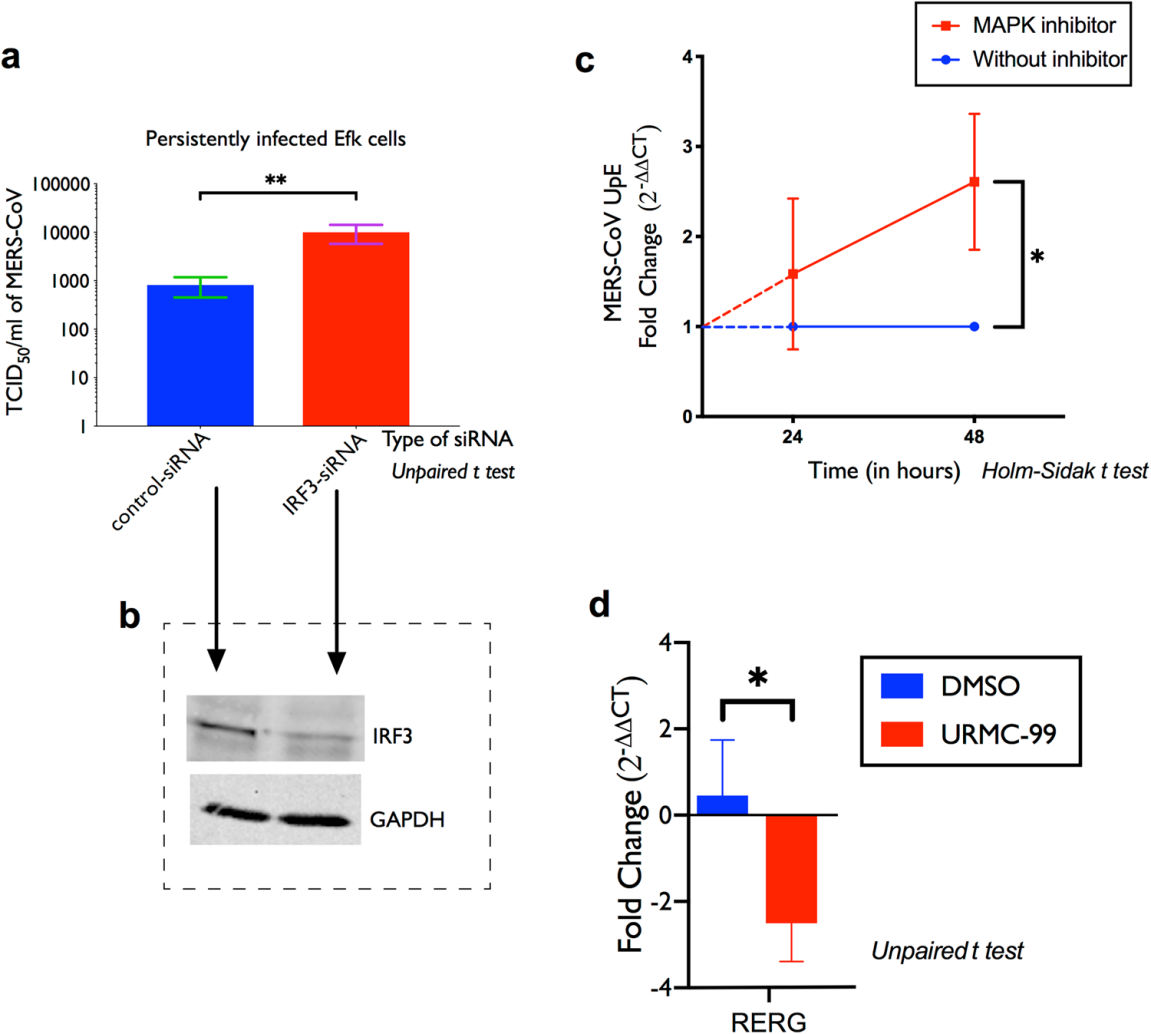
IRF3 and MAP kinase-mediated signaling regulate persistent infection in Efk cells. Persistently infected Efk cells were transfected with siRNA targeting IRF3 mRNA and the subsequent effect on virus replication was measured. (**a)** MERS-CoV titres in persistently infected bat cells 24 hours post treatment with IRF3-siRNA (red bar; n = 4; Mean ± SD). Scrambled siRNA (blue bar; control-siRNA) was used as a negative control. **P = 0.0049 (Unpaired t test with α = 0.05). (**b)** Western blot for IRF3 and GAPDH in Efk cells treated or mock treated with IRF3 siRNA. **(c)** MERS-CoV upE transcript levels in Efk cells after treatment or mock treatment with MAPK inhibitor, URMC-99 for 24 and 48 hours. *P = 0.0053 (*Holm-Sidak t test* with α=0.05). (**d)** RERG transcript levels 48-hours post treatment with URMC-99 (n = 4; Mean ± SD). *P = 0.017 (*Holm-Sidak t test* with α = 0.05). For full size gel images in **(b)**, see supplementary Fig. S3.

Interferon regulatory factor 3 (IRF3) is a central regulator of innate antiviral responses in mammalian cells^36^ and it is involved in the regulation of antiviral responses to MERS-CoV in bat cells^31^. To determine if IRF3-mediated innate antiviral responses play a role in the maintenance of persistent infection in bat cells, we reduced IRF3 protein levels (Fig. 6b) in these cells using small-interfering RNA (siRNA). siRNA-mediated reduction of IRF3 in persistently infected bat cells increased MERS-CoV titers by ten-fold (Fig. 6a). Reducing IRF3 protein levels in persistently infected bat cells also increased cell death within 48 hours of siRNA treatment compared to control siRNA-treated persistently infected cells (data not shown).

In little brown bats (*Myotis lucifugus*) that are co-infected with a bat CoV and White Nose Syndrome-causing fungus (*P. destructans*), suppression of the MAPK pathway has been speculated to increase CoV replication^26^. To test if this pathway played a similar role in regulating viral load in persistently infected bat cells in culture, we inhibited the MAPK signaling pathway using a commercial MAP kinase inhibitor, URMC-99^37^. We observed a significant increase in MERS-CoV replication (upE levels) upon inhibiting the MAPK pathway (Fig. 6c). We confirmed the suppression of the MAP kinase pathway in bat cells by demonstrating a reduction in the transcript levels of a MAPK pathway-related Ras superfamily protein, RAS-like estrogen regulated growth inhibitor (RERG; Fig. 6d)^38,39^.

Since ΔORF5 MERS-CoV was repeatedly selected for in persistently infected Efk cells, we predicted the cellular localization of ORF5 to identify if there was a potential detrimental effect on cells expressing ORF5. By using transmembrane domain prediction softwares and immunofluorescent microscopy, we observed that MERS-CoV ORF5 has 3 transmembrane domains (see Supplementary Fig. S4a) and ectopically expressed MERS-CoV ORF5 localized to the endoplasmic reticulum (ER) in bat cells (see Supplementary Fig. S4b). As bat coronaviruses from the beta-CoV sub-family have ORF5 homologues^18^, we compared MERS-CoV ORF5 with that of ORF5 found in bat beta-CoVs. We found differences in the predicted cytoplasmic domain of MERS-CoV ORF5 (see Supplementary Figs. S4c,d), which may be associated with high toxicity and cell death that we observed with W + MERS-CoV infections in human and bat cells^40^.

## Discussion

Successful spillover of a virus to a new species requires viruses to adapt to the use of viral receptors and circumvent innate antiviral defense mechanisms that are unique to each host species. Due to the lack of well characterized *in vivo* experimental models, it is difficult to examine the processes by which coronaviruses, such as those that cause SARS, MERS, porcine epidemic diarrhea (PED) and swine acute diarrhea syndrome (SADS) have adapted from maintenance in bats to successful infection in their spillover species, such as humans, camels, civet cats and pigs. While closely related coronaviruses have been detected in insectivorous bat species^41,42^, the “culprit” viruses that have caused serious disease in spillover mammalian species have not been isolated from, or characterized in bats.

In this study, we established a long-term persistent infection in bat cells to examine how MERS-CoV (EMC/2012), isolated from a human infection, adapts to replication in cells from an insectivorous bat. Here we demonstrate that unlike in human cells, where infection leads to death of all cells, MERS-CoV establishes a persistent infection in a bat cell line. We observed that: a) Infection of bat cells led to a long-term persistent infection. b) The viability of the cells in culture did not depend on the presence of uninfected resistant cells, as seen in some models of coronavirus persistence^43^. All bat cells in culture contained viral RNA and protein. c) Viral persistence was accompanied by the rapid selection of mutations in the accessory gene – *ORF5*, and this mutation was “fixed” for at least 15 passages of the culture over 4 months. While viral persistence was accompanied by mutations in other genes, none of the mutations appeared to be as drastic as the *ORF5* mutations. The genomic locations of other mutations in MERS-CoV also varied between the two independent attempts at establishing persistence. d) The ΔORF5 mutant virus replicated more efficiently in bat cells than W + MERS-CoV, and cells infected at high MOIs by the ΔORF5 mutant contained higher levels of some interferon-stimulated and anti-apoptotic transcripts. e) Cells persistently infected with the ΔORF5 mutant were resistant to infection by W + MERS-CoV. Compared to uninfected or W + MERS-CoV-infected Efk cells, cells persistently-infected with ΔORF5 MERS-CoV expressed greatly reduced levels of DPP4 transcripts, the known receptor for W + MERS-CoV. f) Persistence was maintained by the retention of higher basal levels of cellular interferon response, as suppression of the response by down-regulating IRF3 increased virus replication.

**Figure 7:**
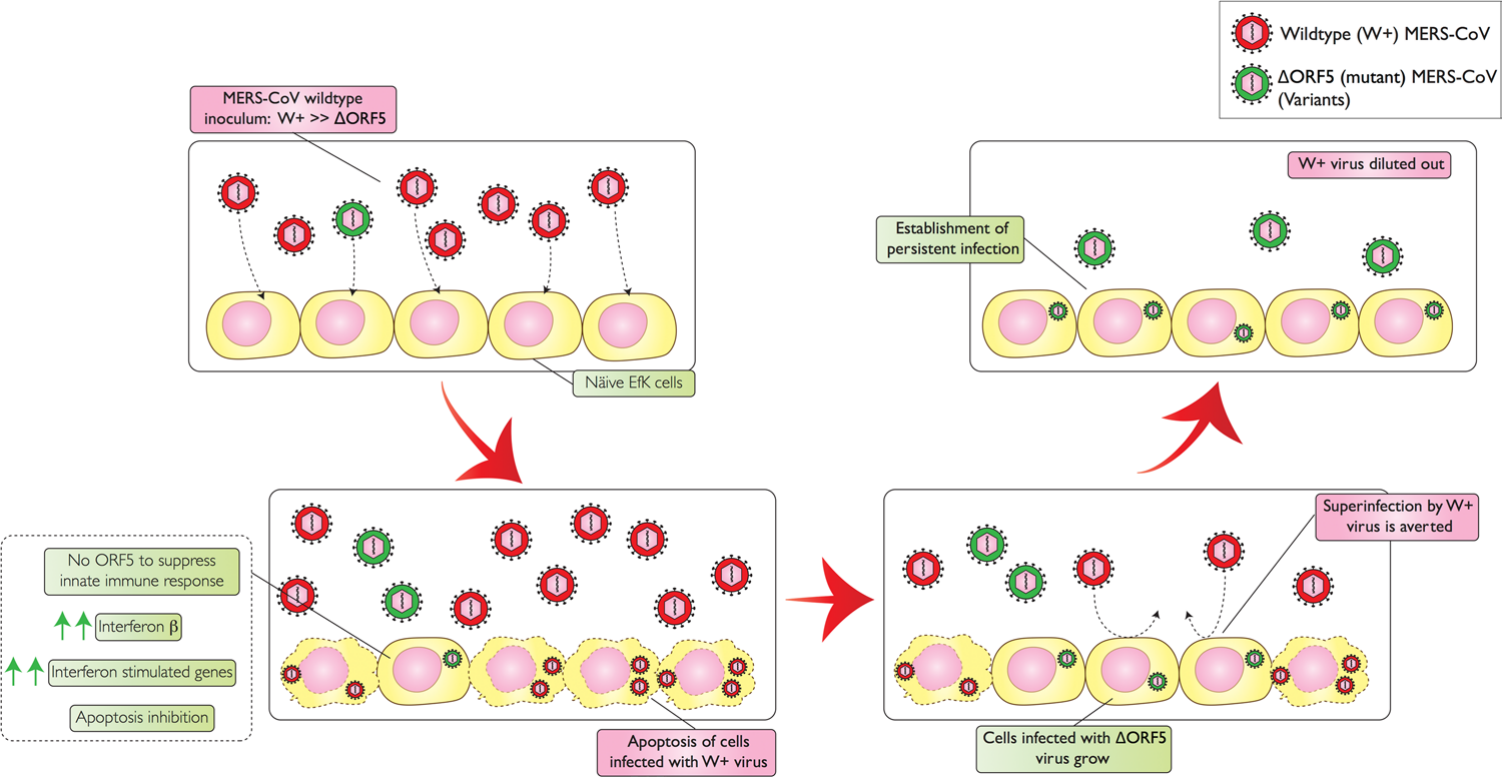
Proposed model for establishment of persistent MERS-CoV infection in bat cells. As with the stocks of most RNA viruses, the MERS-CoV inoculum is made up of the dominant W + virus as well as smaller numbers of variants, including variants with inactivating mutations in ORF5 (ΔORF5). The cells infected with the more cytolytic W + virus die, while the small number of cells infected with ΔORF5 MERS-CoV survive because of an ensuing antiviral response and the induction of anti-apoptotic processes. ΔORF5 MERS-CoV infected cells are resistant to infection with the W + virus and the W + virus is soon diluted out. After a process of cell death and recovery, ΔORF5 MERS-CoV infected cells survive and take over, leading to a culture of persistently infected cells that produce small, but consistent amounts of virus over time.

Our observations lead us to suggest a scenario for the molecular mechanisms that govern persistence of MERS-CoV in bat cells (Fig. 7): MERS-CoV stocks grown in permissive primate (Vero) cells largely comprise W + virus that is cytolytic in both primate and bat cells. Due to the error-prone nature of the viral RNA polymerase, the laboratory stock likely contains variants that are less successful for replication in primate cells than the W + virus. These mutants include some with alterations in the ORF5 gene that render the virus less cytolytic for bat cells. Infection of bat cells at a low MOI leads to the infection of cells by either the W+ or ΔORF5 mutant virus. Due to the cytolytic nature of the W + virus, cells infected by it die with the subsequent release of more W + virus. While cells infected by the less cytolytic ΔORF5 mutants also produce virus, they survive. ΔORF5 mutant virus infected cells survive because the virus induces a more robust innate antiviral response in these cells and the apoptotic processes that accompany W + virus infection are dampened (see IFI6 expression; Fig. 4b). Since infection with ΔORF5 MERS-CoV renders bat cells resistant to infection by W + virus, W + virus is eventually replaced in the culture by ΔORF5 MERS-CoV. Persistence is maintained by elevated levels of basal innate antiviral responses in these cells and suppression of this response leads to an increase in viral replication and cell death.

An interesting observation in our study is the rapid selection of mutations in the gene for ORF5, suggesting that deletion of the coding sequences or other mutations in the gene alter its function in the W + virus and may be an important adaptation for maintenance in bat cells. The role of ORF5 and other accessory genes of beta-coronaviruses has not been clearly established. CoV accessory genes do not appear to be required for virus replication in cultured cells but are thought to be important for infection *in vivo* or for virulence in specific hosts. In human cells, MERS-CoV structural proteins, such as M, and accessory proteins, such as ORFs 4a, 4b and 5 are known to effectively inhibit innate antiviral responses, however, the extensive role of ORF5 in modulating cellular processes has not been investigated^24,44^. Experiments with adapting MERS-CoV to cause disease in humanized mice suggest a role for ORF5 and other accessory viral genes in disease outcome^45,46^. However, the role of ORF5 in MERS-CoV infection of bat cells or bats has not been explored.

An *in-silico* analysis of the W + MERS-CoV ORF5 amino acid sequence revealed three potential trans-membrane regions (see Supplementary Fig. S4a), suggesting that it is a membrane-associated protein. Three trans-membrane accessory proteins in alphacoronaviruses, such as feline infectious peritonitis virus^47^, porcine epidemic diarrhea virus^48^ and human CoV 229E^49^, as well as in betacoronaviruses, such SARS-CoV^50^ also appear to be associated with virulence.

We co-expressed ORF5-linked to red fluorescent protein with Luman-GFP, a green fluorescent protein-linked protein that localizes to the endoplasmic reticulum (ER). The two proteins co-localized in the ER indicating that the protein coded by *ORF5* is associated with the ER (see Supplementary Fig. S4b). Given these data, we have suggested an ER-associated structure of the ORF5 protein with its carboxyl terminal portion in the cytoplasm (see supplementary Fig. S4c). Interestingly, several bat beta coronaviruses also possess an ORF5 homologue. However, the putative cytoplasmic domains of these proteins differ from those of human and camel MERS-CoV isolates at specific residues (see Supplementary Fig. S4d). While the role of MERS-CoV ORF5 in adaptation for replication in different hosts will require further investigation, based upon the reduced cytotoxicity of *ORF5* deletion mutant, we hypothesize that the protein promotes ER-stress induced apoptosis. Viruses with a deleted *ORF5* gene or gene specifying a deleted (or mutated) carboxyl terminus would be less likely to trigger apoptotic cell death in ER-stressed persistently infected cells.

We observed that cells persistently infected with MERS-CoV with deleted or truncated *ORF5* were resistant to superinfection with W + MERS-CoV. This phenomenon may explain the rapid selection of the mutant (ΔORF5 MERS-CoV) and dilution of W + virus. While we have not clearly established the molecular mechanisms that render the persistently infected cells resistant to superinfection, our results indicate that down-regulation of DPP4, the cellular receptor for MERS-CoV (Fig. 5c) in the persistently infected cells provide an explanation. While we detected DPP4 transcripts in the cells (Fig. 5c), the levels in persistently infected cells (relative to a universally expressed house-keeping gene) were about a hundred times lower than in W + MERS-CoV infected naïve Efk cells.

Superinfection exclusion, a phenomenon by which viruses, following infection, exclude infection by similar viruses is well known and occurs in cells persistently or lytically infected with herpesvirus^51,52^, alphaviruses^53^, flaviviruses^54^, papillomaviruses^55^ and influenza viruses^56^. The mechanisms by which infected cells are rendered resistant to further infection vary, but often rely on the occlusion of receptors for the superinfecting virus. We found that the levels of DPP4 transcripts in infected cells did not decrease following infection of Efk cells with either W + or ΔORF5 MERS-CoV (Figs. 4f and 5c). This suggests that during a normal infection at a high MOI, MERS-CoV does not down-regulate the expression of the receptor. However, a lack of bat specific immunological reagents prevented us from detecting bat DPP4 on the surface of MERS-CoV infected Efk cells, so we cannot rule out a decrease in the expression of the protein on the cell surface or occlusion by the viral spike protein.

While persistence in bat cultured cells, devoid of the *in vivo* environment, may not exactly recapitulate MERS-CoV – bat interactions, our model of MERS-CoV persistence mirrors some features observed during enhanced replication of persistently-infecting CoV in bats suffering from White Nose Syndrome^25,26^. Our model also allows us to develop hypotheses that can then be tested by observations in a wild free-living species.

## Materials and Methods

### Cell culture

*Eptesicus fuscus* kidney cells^30^ (Efk or bat cells) were grown in Dulbecco’s Minimal Essential Medium with GlutaGro (DMEM; Corning. Cat #MT10017CV) containing 10% fetal bovine serum (FBS; Sigma, Cat #F0392), penicillin/streptomycin (Gibco, Cat #15140122) and GlutaMax (Gibco, Cat #35050061). Persistently infected Efk cells were maintained and passaged in the same media as Efk cells. Vero 76 (green monkey kidney; ATCC, Cat #CRL-1587) cells were grown in DMEM supplemented with 10% FBS and penicillin/streptomycin. Human lung (MRC5) cells (ATCC CCL-171) were cultured in Minimum Essential Medium Eagle (MEM; Corning) supplemented with 10% FBS, 1/100 non-essential amino acids (NEAA; Gibco), 1/100 4-(2-hydroxyethyl)-1-piperazineethanesulfonic acid (HEPES; Gibco) and 1/1000 gentamycin (Gibco). Cells were incubated in a humidified incubator at 37 °C with 5% CO_2_.

### Virus infection

All work with infectious MERS-CoV was performed in containment level 3 at VIDO-InterVac and was approved by the institutional biosafety committee. Sample inactivation was performed according to standard operating procedures approved by the institutional biosafety committee. MERS-CoV (isolate EMC/2012^57^) was propagated in Vero 76 cells in DMEM (Sigma) supplemented with 10% fetal bovine serum, 50 U/ml penicillin and 50 µg/ml streptomycin (Gibco, Cat #15140122). In the text, low multiplicity of infection (MOI) refers to an infectious dose of 0.01 TCID_50_/cell and high MOI refers to an infectious dose of 10 TCID_50_/cell.

For establishing persistent infection, Efk cells were seeded at a concentration of 3 × 10^5^ cells/well in one well of a six well plate. The cells were infected with MERS-CoV (strain EMC/2012) at a multiplicity of infection (MOI) of 0.01. Growth medium on the cells was replaced regularly and surviving cells were split using 0.25% trypsin (Sigma, Cat #20233). Supernatant from each passage was saved at −80 °C for virus titration. For acute infection, Efk cells were seeded at a concentration of 3 × 10^5^ cells/well in a six well plate. The cells were infected with MERS-CoV (strain EMC/2012) at a multiplicity of infection (MOI) of 10 TCID_50_/ml. ΔORF5 MERS-CoV was rescued by passaging the virus collected from the supernatant of persistently infected Efk cells on Vero 76 cells. The deletion in ORF5 was confirmed by polymerase chain reaction (PCR) and Sanger sequencing.

### Virus titration

Supernatant from persistently infected bat cells were titrated in triplicates on Vero 76 cells using tissue culture infectious dose 50 (TCID_50_) assay. Briefly, 10^4^ cells were seeded in each well of a 96-well plate. The plates were incubated overnight to obtain a confluent layer of Vero 76 cells. The virus sample (supernatant) was diluted ten-fold and 50μl of the diluted virus sample was added to each well of the 96-well plate. The plates were incubated at 37 °C for 1 hr. After incubation, the virus containing supernatant was discarded and 100μl of complete media with 5% FBS was added to the plates. The plates were incubated for 5 days and cytopathic effect was observed under a light microscope. Tissue culture infectious dose 50/ml (TCID_50_/ml) was calculated using the Spearman and Karber algorithm^58,59^.

### Immunoblot

Persistently infected Efk cells were seeded at a concentration of 3 × 10^5^ cells/well in six well plates and incubated at 37 °C overnight. Next day, cells were harvested in sample buffer for western blots. Western blots were carried out as previously mentioned^60^. Briefly, samples were denatured in a reducing sample buffer and electrophoresed on a reducing gel. Proteins were blotted from the gel onto polyvinylidene difluoride (PVDF, GE Healthcare, Cat #10600023) membranes and detected using primary and secondary antibodies. Primary antibodies used were: 1: 1,000 rabbit anti-IRF3 (Abcam; Cat# ab68481), 1: 1,000 mouse anti-GAPDH (EMD Milipore, Cat #AB2302) and 1: 1,000 rabbit anti-MERS-CoV N protein (Sino Biological, Cat #40068-RP01). Secondary antibodies used were: 1:10,000 goat anti-mouse Alexa 488 (Molecular Probes, Cat #A-11001) and 1: 10,000 goat anti-rabbit Cy5 (GE Healthcare, Cat #PA45012). Blots were observed and imaged using a Typhoon Scanner (Amersham Biosciences).

### Electron microscopy

Persistently infected Efk cells were scraped in phosphate buffered saline and pooled in a 15 ml screw-cap centrifuge tube. The cells were recovered by centrifugation at 300xg for 5 mins and the supernatant was discarded. The cell pellet was resuspended in 10% neutral buffered formalin (10% NBF; Sigma, Cat #HT501128) for virus inactivation. The cells were later processed for electron microscopy as mentioned previously^25^. Briefly, cells were treated with osmium tetroxide (1% OsO_4_, 0.1 M sodium cacodylate buffer) for one hour at room temperature. Samples were quickly rinsed with water, gradually dehydrated in ethanol and *en*-bloc stained with uranyl acetate. After rinsing three times (5 min each) in propylene oxide, samples were infiltrated with Epon/Araldite (electron Microscopy Sciences, Cat #50-980-381). Samples were placed in molds and freshly prepared Epon/Araldite was added. The samples were then polymerized at 60 °C for 24–48 h. Sections of 90 nm were cut and observed by transmission electron microscopy (TEM - Hitachi HT 7700, Tokyo, Japan).

### Immunofluorescence

Persistently infected and mock/acute infected Efk cells were seeded at a concentration of 3 × 10^4^ cells/chamber in chamber slides (Nunc, Thermo Scientific, Cat #154534). Each chamber was harvested at the respective time points by removing media, a quick rise with PBS and addition of 10% NBF. 10% NBF was added to inactivate and fix the cells over a period of 48 h. After removing the cells from containment, cells in each chamber were washed twice with phosphate buffered saline (PBS). The slides were permeabilized using 0.2% TritonX-100 (VWR, 97062–208) diluted in PBS for 5 minutes. Cells were incubated in a blocking solution [PBS, 10% donor calf serum (Sigma, Cat #C9676) and 0.1% Tween 20 (Fisher Bioreagents, Cat #BP337–500)]. Primary staining for MERS-CoV nucleoprotein (N) and GAPDH was performed using 1:100 dilution of rabbit anti-MERS-CoV N (Sino Biological, Cat #40068-RP01) and mouse anti-GAPDH (EMD Milipore, Cat #AB2302). Secondary staining was performed using 4 μg/ml goat anti-mouse Alexa 488 (Molecular Probes, Cat #A-11001), 0.1 μg/ml goat anti-rabbit Cy5 (GE Healthcare, Cat #PA45012) and 0.2 μg/ml Hoechst 33342 (Molecular Probes, Cat #H3570) in blocking solution. For ORF5-RFP and Luman-GFP (endoplasmic reticulum marker) constructs, cells were co- transfected and observed under the microscope after overnight incubation. Cells were observed under an Olympus IX83 fluorescence microscope.

### *In-situ* hybridization

As for immunofluorescence, persistently infected and mock/acute infected Efk cells were seeded at a concentration of 3 × 10^4^ cells/chamber in chamber slides (Nunc, Thermo Scientific). Each chamber was harvested at the respective time points by removing media, a quick rinse with PBS and addition of 10% NBF. 10% NBF was added to inactivate and fix the cells over a period of 48 h. MERS-CoV RNA was detected in the cells using ViewRNA ISH tissue 2-plex assay kit (Affymetrix, Thermofischer Scientific, Cat #QVT0012) and probes targeting MERS-CoV nucleocapsid sequence (Thermofischer Scientific, United States) as per manufacturer’s instructions, with the exception of xylene treatment of slides, which was omitted. Counterstaining of cells was done using Gill’s hematoxylin I (Sigma Aldrich, Cat #GHS-132). Coverslips were applied at the end of the assay and images were taken using brightfield microscope with 20X and 40X objectives.

#### RNA extraction, cDNA preparation and quantitative real-time PCR (qRT-PCR)

 All RNA extractions were performed using the RNeasy Plus Mini kit (Qiagen, Cat #74136) as per manufacturer’s instructions. cDNA was prepared using iScript gDNA clear kit (Bio-Rad) as per manufacturer’s instructions. 500 ng of RNA was used for cDNA preparation. cDNA was used as a template for the quantification of target genes. Quantitative real-time polymerase chain reaction (qRT-PCR) assays amplifying targeted cellular genes and the normalizer gene (Glyceraldehyde-3-phosphate; GAPDH) were performed for both MRC5 and Efk cells. Primer sequences are listed in Table 2. MERS-CoV gene primers were limited in their capacity to detect genomic and sub-genomic RNA pertaining to the respective gene segment. For example, nucleocapsid primer was designed to detect nucleocapsid gene segment present in the genomic RNA as well as in the sub-genomic RNA. Primers for quantifying GBP1, IL8, Mx1, IRF3, MDA5, IRF7, TNFα and IFI6 were used as mentioned previously^61^. Bio-Rad’s CFX96 Touch PCR thermocycler was used in conjunction with Bio-Rad’s Ssofast Evagreen supermix (Bio-Rad, Cat #1725204) and samples were prepared as previously described^62^. For qRT-PCR, after the initial denaturation step of 95 °C for 5 minutes, two step cycling for 40 cycles was performed at 95 °C/10 s, 56 °C/30 s. Absorbance readings were acquired after each cycle. The final three steps were carried out at 95 °C/1 min, 55 °C/30 s and 95 °C/30 s to generate the dissociation curve. Absorbance readings for the dissociation curve were acquired at every degree from 55–95 °C. Relative fold change in gene expression was calculated after normalizing the Ct values using GAPDH and the data was normalized to mock infected samples or as mentioned in the figure legends. Difference of one Ct indicates a two-fold difference in gene expression. Graphs were plotted using GraphPad prism version 7.

**Table 2:** Sequences of siRNA and primers used for qPCR/conventional PCR.

Target gene	Forward primer (5′-3′)	Reverse primer (5′-3′)
upE	GCAACGCGCGATTCAGTT	GCCTCTACACGGGACCCATA
S	TGGTCTTTGCGATGCAGCTA	TTGGAAGTCAATCCCGGTGG
ORF3	TGAGAGTTCAAAGACCACCCAC	TGATTCTGCAGATGGGACGT
ORF4a	GCACTTCATTGCACCCTGTG	TGTAGCAACCAAGCGATTCG
ORF4b	TTCTGCGCCATGAAGACCTT	GGCCGCCATAAGGTTTAAGC
ORF5	TGTTTGACATGCGTTCCCAC	TTGCAGGCACGAAAACAGTG
E	ATGTTACCCTTTGTCCAAGAAC	TTAAACCCACTCGTCAGGTGG
M	TCGGTGCTTGTGACTACGAC	CCGTAATAGGCGGACTCCTG
N	ACCACGAGCTGCACCAAATA	ATGGACCCAAACGATGCCAT
IFNβ	GCTCCGATTCCGACAGAGAAGCA	ATGCATGACCACCATGGCTTC
GAPDH	GGAGCGAGATCCCGCCAACAT	GGGAGTTGTCATACTTGTCATGG
Ef DPP4	TGATCTTGCCTCCTCATTTTGATAA	GTAACCACTTCCTCTGCCATCAA
Hu DPP4	TGA CAT GGG CAA CAC AAG A	AAC CCA GCC AGT AGT ACT C
DPP4 (consensus primers; human and *E. fuscus*)	CAC CAT CAT CAC CGT GCC C	CAA ATG TAC TGT TCT CC
ORF5 (Full length)	GCCGGTACCATGGCTTTCTCG	GCCGGATCCTTACGATAAGCG
mRFP	GCCGGATCCAAATGGCCTCCTCCGAG	GCCCTCGAGTTAGGCGCCGGTGGA
siRNA-IRF3–1 (duplex)	5′ rCrArArGrArArGrCrUrArGrUrGrArUrGrGrUrCrArArGrGTT 3′	
	5′ rArArCrCrUrUrGrArCrCrArUrCrArCrUrArGrCrUrUrCrUrUrGrGrU 3′	
siRNA-IRF3–2 (duplex)	5′ rCrUrGrCrCrArArCrCrUrGrGrArArGrArGrGrArArUrUrUCA 3′	
	5′ rUrGrArArArUrUrCrCrUrCrUrUrCrCrArGrGrUrUrGrGrCrArGrGrU 3′	

### IRF3 knockdown and MAP kinase inhibition

Dicer-ready siRNA (DsiRNA) specific to big brown bat and human IRF3 was designed and obtained through Integrated DNA Technologies (IDT). A 100 nM final concentration of a 1:1 mixture of two DsiRNAs per cell line (Table 2) targeting separate regions on the big brown bat IRF3 transcript was transfected into persistently infected Efk cells using Lipofectamine 2000 (Invitrogen, Cat #11668019). Scrambled non-specific DsiRNA (NC DsiRNA; IDT) was used as a negative control. For MAP kinase inhibition, URMC-099 drug that was resuspended in DMSO (Cayman chemicals, Cat #1229582-33-5) was used at a concentration of 1 µM^63^. DMSO only was used as a negative control. Cells were harvested after 24 and 48 hours and virus replication was quantified using MERS-CoV UpE primers and qPCR.

### Next generation sequencing

For Sanger sequencing of ORF5, RNA was extracted from MERS-CoV persistently-infected Efk cultures using QIAamp Viral RNA Mini Kit (QIAGEN) following the manufacturer’s protocol. cDNA was synthesized using Superscript III Reverse Transcriptase (Invitrogen) and random hexamers. MERS-CoV genome was amplified in 1.5–2.5 kB overlapping fragments using iProof High Fidelity DNA Polymerase (Bio-Rad Laboratories, Inc.). PCR products were purified with a QIAquick PCR Purification Kit (QIAGEN) and used as input for the Illumina Nextera DNA Flex Library Prep Kit (Illumina, Inc.). Libraries were sequenced with 150 bp paired-end reads on Illumina MiSeq. Reads were mapped to the reference genome using BWA-MEM^64^.

### Cloning

Conventional PCR was carried out to amplify full-length MERS-CoV ORF 5 with restriction sites KpnI and BamHI using cDNA prepared from RNA that was extracted from Efk cells infected with MERS-CoV. Red fluorescent protein (RFP) with restriction sites BamHI and XhoI was amplified from pcDNA3-mRFP using mRFP primers. PCR was performed using the following thermal cycle profile: initial denaturation for 3 min at 94 °C, 35 PCR cycles at 94 °C/30 s, 56 °C/30 s and 72 °C/1 min. The final extension was at 72 °C for 10 min. The two PCR products were ligated into a pcDNA3 backbone that was digested with KpnI and XhoI. Plasmid resulting from the double ligation was used to transform DH5α *E. coli* cells. Bacterial cells were plated on 2YT plates with ampicillin. Resistant bacterial colonies were scanned for plasmids carrying the ORF5-RFP insert. Positive clones were amplified in broth culture. Plasmids were later purified and concentrated using a MaxiPrep kit (QIAGEN, Cat #12163). The endoplasmic reticulum marker, Luman-GFP was made in a previous study (53–55).

### ORF5 domain prediction and multiple sequence alignment

ORF5 domain prediction was performed by inputting the ORF5 amino acid sequence into the TMHMM Server v.2.0^65^. Amino acid sequence from CoVs were retrieved from NCBI’s database (accession numbers in supplementary Fig S4d), and multiple sequence alignment was performed using MacVector software^66^.

### Statistics and softwares

Changes in the mean was analyzed by *Holm-Sidak t test* for multiple group comparison (time point experiments) and *P* < 0.05 was considered significant. For two group comparisons, we used *unpaired t test* and *P* < 0.05 was considered significant. All statistical analyses and graphs were generated using GraphPad Prism v8. Schematic diagrams were made using Adobe Illustrator v24.0.1. Transmembrane domain prediction and multiple sequence alignments were performed using TMHMM Server v2.0 and MacVector v17.5^65,66^.

## Supplementary information

Supplementary Information.
