# Supplementary material for: Selection of viral variants during persistent infection of insectivorous bat cells with Middle East respiratory syndrome coronavirus
Source: Sci Rep. 2020 Apr 29;10:7257. doi: 10.1038/s41598-020-64264-1 (PMC7190632; doi:10.1038/s41598-020-64264-1)
Supplement: Supplementary file 1 — Supplementary Information. [file 41598_2020_64264_MOESM1_ESM.docx]

# Supplemental Information

# Persistent infection of bat cells with Middle East respiratory syndrome (MERS) coronavirus selects for viral variants

*Arinjay Banerjee^Ψ,^ ^1,#^, Sonu Subudhi^Ψ,^ ^1,$^, Noreen Rapin^1^, Jocelyne Lew^2,^, Richa Jain^2^, Darryl Falzarano^1,2,φ^ and Vikram Misra^1,φ ,*.^*

^1^Department of Veterinary Microbiology, Western College of Veterinary Medicine, University of Saskatchewan, Saskatoon, SK, Canada.

^2^Vaccine and Infectious Disease Organization-International Vaccine Centre (VIDO-InterVac), Saskatoon, SK, Canada.

^#^Present address: Department of Pathology and Molecular Medicine, Michael DeGroote Institute for Infectious Disease Research, McMaster University, Hamilton, ON, Canada.

^$^Present address: Gastrointestinal Unit and Liver Center, Massachusetts General Hospital, Harvard Medical School, Boston, MA, USA

*Corresponding author: Prof. Vikram Misra

Email: vikram.misra@usask.ca

*^Ψ^ Contributed equally to the manuscript*

*^φ^ Principal investigators*

**Supplementary Figures**

**Figure S1. (a-c)Electron micrographs showing virus particles (red arrows) in persistently infected Efk cells.**

**Figure S2. Related to Figure 5c**. Full size gel image of DPP4 detection in Efk cells infected with W+ MERS-CoV and persistently infected Efk cells infected with W+ MERS-CoV.

**
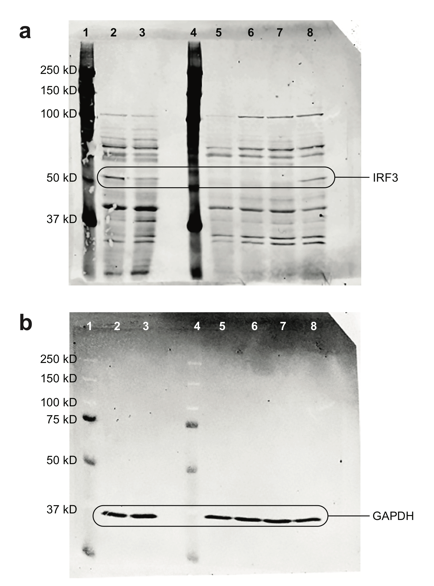
**

**Figure S3. Full sized blots for Figure 6b. (a)** Full blot stained with anti-IRF3 antibody. **(b)** Full blot stained with anti-GAPDH antibody. Lane 1 – protein ladder, lane 2 – control siRNA treated persistently infected Efk cells and lane 3 – IRF3 siRNA treated persistently infected Efk cells. Lanes 4 – 8 were published as part of a different study ^1^. Lane 4 – protein ladder, lane 5 – cr2-9 (IRF3 knockout Efk clonal cell line), lane 6 – cr2-12 (IRF3 knockout Efk clonal cell line), lane 7 – cr3-8 (IRF3 knockout Efk clonal cell line) and lane 8 – wildtype Efk cells.

**Figure S4. Coronavirus ORF5 sequences differ in their C-terminus region and MERS-CoV ORF5 localizes in the endoplasmic reticulum of bat cells. (a)** MERS-CoV ORF5 protein domains predicted using TMHMM Server (version 2.0 - <http://www.cbs.dtu.dk/services/TMHMM/>). **(b)** Immunofluorescent microscopy images showing co-localization of MERS-CoV ORF5-tagged with red fluorescent protein (MERS-ORF5; red) and an endoplasmic reticulum (ER) marker, Luman, tagged with green fluorescent protein (ER marker; green) in Efk cells. **(c)** Relevant amino acid differences between MERS-CoV ORF5 and a related bat CoV ORF5 (NCBI Accession # YP_009361861). **(d)** Multiple sequence alignment of ORF5 C-termini of bat and human coronaviruses. Figure generated using MacVector (version 17.5 - <https://macvector.com>).

**References**

1 Banerjee, A., Falzarano, D., Rapin, N., Lew, J. & Misra, V. Interferon Regulatory Factor 3-Mediated Signaling Limits Middle-East Respiratory Syndrome (MERS) Coronavirus Propagation in Cells from an Insectivorous Bat. *Viruses* **11**, doi:10.3390/v11020152 (2019).
